# *BRAF *mutations are associated with distinctive clinical, pathological and molecular features of colorectal cancer independently of microsatellite instability status

**DOI:** 10.1186/1476-4598-5-2

**Published:** 2006-01-10

**Authors:** Wei Qi Li, Kazuyuki Kawakami, Andrew Ruszkiewicz, Graeme Bennett, James Moore, Barry Iacopetta

**Affiliations:** 1 School of Surgery and Pathology, University of Western Australia, Nedlands, WA 6009, Australia; 2 Department of Surgery, Kanazawa University School of Medicine, Takaramachi 13-1, Kanazawa 920-8641, Japan; 3 Division of Tissue Pathology, Institute of Medical and Veterinary Science, Frome Rd, Adelaide, SA 5000, Australia; 4 Division of Molecular Pathology, Institute of Medical and Veterinary Science, Frome Rd, Adelaide, SA 5000, Australia; 5 Colorectal Surgery Unit, Royal Adelaide Hospital, Adelaide, SA 5000, Australia

## Abstract

**Background:**

BRAF is a member of RAF family of serine/threonine kinases and mediates cellular responses to growth signals through the RAS-RAF-MAP kinase pathway. Activating mutations in *BRAF *have recently been found in about 10% of colorectal cancers, with the vast majority being a V600E hotspot mutation. The aim of the present study was to evaluate the clinical, pathological and molecular phenotype of colorectal tumors with *BRAF *mutations.

**Results:**

Mutations in *BRAF *were identified in 8% (23/275) of colorectal cancers. They were 5–10-fold more frequent in tumors with infiltrating lymphocytes, location in the proximal colon, poor histological grade and mucinous appearance (*P *< 0.002 for each). Tumors with *BRAF *mutation were also 10-fold more likely to show microsatellite instability and frequent DNA methylation (*P *< 0.0001) compared to tumors without this mutation. The characteristic morphological features of tumors with *BRAF *mutation (infiltrating lymphocytes, poor grade, mucinous) remained after stratification according to microsatellite instability and methylator phenotypes. Mutations in *BRAF *were mutually exclusive with mutations in *KRAS *but showed no clear association with the presence of *TP53 *mutation.

**Conclusion:**

*BRAF *mutation identifies a colorectal cancer subgroup with distinctive phenotypic properties independent of microsatellite instability status and thus could be a valuable marker for studies into the clinical properties of these tumors.

## Background

*BRAF *is a member of the RAF family of kinases that acts upstream of the MEK1/2 kinases in response to RAS signals. Activating mutations in *BRAF *have been reported in 5–15% of colorectal carcinomas (CRC), with by far the most common mutation being a 1796T to A transversion leading to a V600E substitution [1-3]. The *BRAF *V600E hotspot mutation is strongly associated with the microsatellite instability (MSI+) phenotype but is mutually exclusive with *KRAS *mutations [4-7]. Interestingly, *BRAF *mutations are found only in MSI+ sporadic tumors that result from aberrant *MLH1 *promoter methylation and do not occur in MSI+ tumors from hereditary non-polyposis colorectal cancer (HNPCC) patients [5,8-10], thus providing a convenient discriminator between sporadic and familial cases. The majority of MSI+ sporadic tumors belong to a larger CRC group referred to as the CpG island methylator phenotype (CIMP+) that is characterised by widespread hypermethylation of CpG islands located with gene promoter regions [11]. Both MSI+ and CIMP+ tumors are thought to arise from large hyperplastic polyps and serrated adenomas [12,13] and recent work has demonstrated a high frequency of *BRAF *mutations in these lesions [7,14,15].

Although the positive association with MSI+ and inverse association with *KRAS *mutation have been well documented, little is known about the other properties of tumors with *BRAF *mutation. In the present study we analysed for *BRAF *V600E mutations in a consecutive series of 275 CRCs that were well characterised for the major pathological and molecular features of this disease. Our results demonstrate that oncogenic *BRAF *mutation occurs preferentially within a subgroup of CRCs that have distinctive features. It could therefore be used as a convenient marker for the further characterisation of these tumors, particularly in relation to their prognosis and response to adjuvant chemotherapy.

## Results

**Figure 1:**
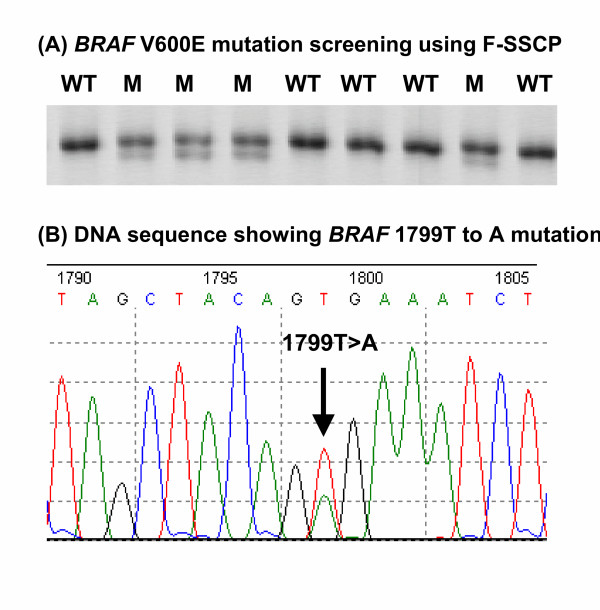
(A) Representative F-SSCP gel used to detect *BRAF *mutationsin colorectal cancer. WT, wild-type; M, mutation. (B) DNA sequencing gel resultconfirms the presence of a 1799T to A mutation giving rise to the V600E mutation.

Figure 1A shows representative Fluorescent-SSCP results for the screening of *BRAF *mutations in this CRC series, while Figure 1B shows DNA sequencing confirmation of the 1799T to A transversion resulting in the V600E mutation. The overall frequency of *BRAF *mutation was 8.4% (23/275), comparing favourably with frequencies of 9–11% reported for other large studies of this tumor type [6,16,17]. The mean age of patients with and without *BRAF *mutation was identical (Table 1). Strong associations were observed between *BRAF *mutation and tumor origin in the proximal side of the large bowel, poor histological grade, mucinous appearance and the presence of infiltrating lymphocytes. Higher frequencies of *BRAF *mutation were also observed in females and in node negative tumors but these did not reach significance.

**Table 1:** Associations between *BRAF *mutation and clinicopathological features of colorectal cancer.

**Feature (n) ^a^**	***BRAF *wild-type (%)**	***BRAF *mutation (%)**	***P***
Total (275)	252 (92)	23 (8)	
			
Age (yrs)	68.4 ± 13.0	68.4 ± 20.7	NS
			
Gender			
Men (132)	124 (94)	8 (6)	
Women (100)	87 (87)	13 (13)	0.068
			
Infiltrating lymphocytes			
Negative (199)	190 (95)	9 (5)	
Positive (21)	11 (52)	10 (48)	<0.0001
			
Nodal involvement			
Negative (128)	115 (90)	13 (10)	
Positive (70)	66 (94)	4 (6)	NS
			
Tumor site			
Proximal (93)	79 (85)	14 (15)	
Distal (126)	122 (97)	4 (3)	0.0015
			
Histological grade			
Well/moderate (140)	133 (95)	7 (5)	
Poor (29)	22 (76)	7 (24)	0.0006
			
Mucinous			
Negative (159)	150 (94)	9 (6)	
Positive (27)	20 (74)	7 (26)	0.0005

^a ^Data was unavailable for gender in 43 cases, infiltrating lymphocytes in 55 cases, nodal involvement in 77 cases, tumor site in 56 cases, grade in 106 cases and mucinous appearance in 89 cases.

*BRAF *mutations showed no association with *TP53 *mutations and were mutually exclusive with the presence of *KRAS *mutations (Table 2). In contrast, *BRAF *mutations were approximately 10-fold more frequent in MSI+ and CIMP+ tumors compared to tumors without these phenotypes. A strong association was also seen with methylation of the *MLH1 *gene promoter and in particular with methylation of its proximal region. We have previously examined the methylation status of 7 different CpG islands in this CRC series [18]. The mean number of these methylated sites was 3-fold higher in tumors with *BRAF *mutation compared to those without (2.6 ± 1.7 *vs *0.8 ± 1.0; *P *< 0.001). Multivariate analysis revealed that MSI+ was the only significant independent predictor of *BRAF *mutation (RR = 6.3, 95%CI [1.2–32.3]; *P *= 0.028) in a model that included CIMP+, tumor site, histological grade, presence of infiltrating lymphocytes and mucinous appearance.

**Table 2:** Associations between *BRAF *mutation and molecular features of colorectal cancer.

**Feature (n) ^a^**	***BRAF *wild-type (%)**	***BRAF *mutation (%)**	***P***
Total (275)	252 (92)	23 (8)	
			
MSI			
negative (204)	195 (96)	9 (4)	
positive (31)	19 (61)	12 (39)	<0.0001
			
Methylation status			
CIMP- (150)	145 (97)	5 (3)	
CIMP+ (42)	31 (74)	11 (26)	<0.0001
			
*MLH1 *distal region			
Negative (168)	159 (95)	9 (5)	
Positive (24)	17 (71)	7 (29)	<0.0001
			
*MLH1 *proximal region			
Negative (179)	169 (94)	10 (6)	
Positive (13)	7 (54)	6 (46)	<0.0001
			
*KRAS*			
Wild-type (156)	134 (86)	22 (14)	
Mutant (93)	93 (100)	0 (0)	<0.0001
			
*TP53*			
Wild-type (183)	166 (91)	17 (9)	
Mutant (66)	62 (94)	4 (6)	NS

^a ^Data was unavailable for MSI status in 40 cases, methylation status in 83 cases, *KRAS *mutation in 26 cases and *TP53 *mutation in 26 cases

We next examined whether the characteristic features of tumors with *BRAF *mutation were still apparent following stratification into MSI and CIMP phenotypes. Although the statistical power of this subgroup analysis was limited, the morphological features of infiltrating lymphocytes, poor histological grade and mucinous appearance were clearly associated with *BRAF *mutation regardless of tumor MSI status (Table 3). Similarly, these features were each more common in tumors with *BRAF *mutation in both the CIMP- and CIMP+ subgroups (Table 4). Similar to previous observations in a separate CRC cohort [20], the frequency of *KRAS *mutation was lower in MSI+ compared to MSI- tumors (*P *= 0.034; Table 3), while the frequency of *TP53 *mutation was also considerably lower in MSI+ tumors with wildtype *BRAF *than in MSI- tumors with wildtype *BRAF *(*P *= 0.014).

**Table 3:** Clinicopathological and molecular features of *BRAF *mutant colorectal cancers stratified according to microsatellite instability status.

	**MSI-**			**MSI+**		
				
**Feature**	***BRAF *WT **(n = 192) (%)	***BRAF *M **(n = 9) (%)	***P***	***BRAF *WT **(n = 19) (%)	***BRAF *M **(n = 12) (%)	***P***
Age (years)	68.5 ± 12.6	58.2 ± 26.5	NS	67.5 ± 16.8	76.1 ± 10.9	NS
Females	39	44	NS	63	75	NS
TILS + ^a^	3	44	0.0004	28	60	0.08
Node negative	62	62	NS	81	89	NS
Proximal site	36	67	0.05	72	89	NS
Poor grade	15	40	0.12	25	56	0.11
Mucinous	12	53	0.05	6	44	0.04
CIMP+	15	50	0.03	40	88	0.03
Dist. *MLH1 *methylated	7	12	NS	40	75	0.10
Prox. *MLH1 *methylated	1	0	NS	33	75	0.06
*KRAS *mutant	43	0	0.008	21	0	0.12
*TP53 *mutant	29	11	NS	5	18	NS

^a^Tumor-infiltrating lymphocytes

**Table 4:** Clinicopathological and molecular features of *BRAF *mutant colorectal cancers stratified according to methylator phenotype status.

	**CIMP-**			**CIMP+**		
				
**Feature**	***BRAF *WT **(n = 145) (%)	***BRAF *M **(n = 5) (%)	***P***	***BRAF *WT **(n = 31) (%)	***BRAF *M **(n = 11) (%)	***P***
Age (years)	68.3 ± 13.5	71.0 ± 11.0	NS	71.7 ± 11.8	65.4 ± 26.3	NS
Females	37	60	NS	42	45	NS
TILS +	2	40	0.008	17	45	0.06
Node negative	63	60	NS	65	82	NS
Proximal site	35	60	NS	74	80	NS
Poor grade	13	66	0.05	20	40	NS
Mucinous	9	25	NS	25	45	NS
MSI+	6	20	NS	19	64	0.01
Dist. *MLH1 *methylated	3	0	NS	42	64	NS
Prox. *MLH1 *methylated	0	0	NS	23	55	0.05
*KRAS *mutant	43	0	0.06	55	0	0.001
*TP53 *mutant	26	0	NS	29	20	NS

## Discussion

The *BRAF *V600E mutation has already been proposed as a convenient marker to discriminate between MSI+ tumors that are sporadic or HNPCC in origin [5,8-10]. This is a very important issue for population-based screening programs that aim to identify CRC associated with the HNPCC syndrome. Compared to the analysis of *MLH1 *promoter methylation, mutation at the *BRAF *V600E hotspot is relatively simple to detect using DNA sequencing, RFLP or the SSCP method used in the present work (Figure 1).

Similar to other studies [4,5,10,16,17] we observed *BRAF *mutation frequencies of 4% in MSI- tumors and 39% in MSI+ tumors (Table 1). The highest frequencies were seen in tumors showing methylation of the *MLH1 *promoter proximal region (46%) and in tumors with infiltrating lymphocytes (48%). *BRAF *mutation frequencies of up to 70–80% have been reported in sporadic MSI+, CIMP+ and *MLH1*-methylated CRC and polyps [7,8,15,16]. For reasons that are still unclear, *BRAF *mutations are approximately 5–10-fold more frequent in tumors that have characteristic features of sporadic MSI+ (ie. *MLH1 *methylated) and CIMP+ phenotypes. These include proximal colon location, poor differentiation, mucinous histology and infiltrating lymphocytes [13,19,20]. Interestingly however, in the present study *BRAF *mutations never occurred in association with *KRAS *mutation, were present in only 3% of CIMP- tumors and showed no association with *TP53 *mutation (Table 2). The observation that *BRAF *mutations occur only very rarely in HNPCC-related MSI+ CRC demonstrates that defective DNA mismatch repair is not involved in causing this genetic alteration.

In order to determine whether the characteristic clinicopathological features of tumors with *BRAF *mutation were due to their close association with MSI+ and CIMP+, we stratified tumours according to these phenotypes. Despite having only 9 MSI-/*BRAF *mutant and 5 CIMP-/*BRAF *mutant tumors, the results showed that associations between *BRAF *mutation and the morphological properties of tumor-infiltrating infiltrating lymphocytes, poor histological grade and mucinous phenotype were retained (Tables 3 and 4).

The frequencies of *BRAF *mutation observed in MSI- (4%) and MSI+ (39%) tumors in the present study compare favourably (5% and 52%, respectively) to those reported recently in another large, population-based study [17]. Although *BRAF *mutations are much more frequent in MSI+ tumors, the comparative rarity of this phenotype means that a considerable proportion occur in MSI- tumors. In the present study, 43% of all *BRAF *mutations occurred in MSI- tumors compared to 48% in the study by Samowitz *et al *[17]. *BRAF *mutations were reported to show prognostic significance in MSI- but not in MSI+ CRC [17]. The lack of follow-up information on CRC patients in the current study and the small number of *BRAF *mutations (n = 21) meant that we were unable to evaluate the prognostic significance of *BRAF *mutation according to MSI status.

## Conclusion

Findings from the present study and from previous work indicate that *BRAF *mutation is likely to be a convenient marker for the identification of a subset of CRCs with distinctive clinical, pathological and molecular features and which may originate in hyperplastic polyps and serrated adenomas [7,14,15]. In view of the strong associations between *BRAF *mutation and specific pathological (site, grade, mucinous, infiltrating lymphocytes) and molecular (methylated MSI+, CIMP+, wildtype *KRAS*) features, it will be interesting in future studies to determine the predictive significance of this marker for response to adjuvant therapies in CRC.

## Methods

The 275 colorectal tumors investigated in this study were obtained from the Colorectal Unit of the Royal Adelaide Hospital. These were snap frozen in liquid nitrogen within 20–40 min after resection and stored at -70°C prior to extraction of DNA. Clinical data available for this series included patient age, sex and family history of CRC. Only one case was confirmed as HNPCC-related. Pathological data included nodal involvement, tumor site, histological grade, mucinous appearance and the presence of infiltrating lymphocytes. Evaluation of MSI+ [21], CIMP+ [18], *KRAS *mutation [22] and *TP53 *mutation [23] were performed as described previously by our group. Mutations in exon 15 of *BRAF *including the V600E hotspot were detected using the PCR primer sequences reported earlier [1], the F-SSCP method [22,23] and confirmed by direct sequencing.

Statistical analyses were performed using SPSS Version 12.0 (Chicago, Illinois, USA). Associations between *BRAF *mutation and clinical, pathological or molecular features were evaluated using Fisher's exact or Pearson's chi-squared tests as appropriate. Multivariate analysis was performed using binary logistic regression with *BRAF *mutation as the dependent variable.

## List of abbreviations

Colorectal carcinoma, CRC; microsatellite instability, MSI+; hereditary non-polyposis colorectal cancer, HNPCC; CpG island methylator phenotype, CIMP+; fluorescent single strand conformation polymorphism, F-SSCP; tumor-infiltrating lymphocytes, TILs.

## Authors' contributions

WL analysed for *BRAF *mutations using SSCP. KK carried out the methylation analyses. AR characterized the tumor series for pathological features. GB carried out the analysis for MSI+ status and DNA sequencing of *BRAF*. JM was largely responsible for establishment of the tumor bank. WL, KK, AR and BI analysed and interpreted the data and BI prepared the manuscript. All authors read and approved the final version of the manuscript.

## References

[B1] Davies H, Bignell GR, Cox C, Stephens P, Edkins S, Clegg S, Teague J, Woffendin H, Garnett MJ, Bottomley W, Davis N, Dicks E, Ewing R, Floyd Y, Gray K, Hall S, Hawes R, Hughes J, Kosmidou V, Menzies A, Mould C, Parker A, Stevens C, Watt S, Hooper S, Wilson R, Jayatilake H, Gusterson BA, Cooper C, Shipley J, Hargrave D, Pritchard-Jones K, Maitland N, Chenevix-Trench G, Riggins GJ, Bigner DD, Palmieri G, Cossu A, Flanagan A, Nicholson A, Ho JW, Leung SY, Yuen ST, Weber BL, Seigler HF, Darrow TL, Paterson H, Marais R, Marshall CJ, Wooster R, Stratton MR, Futreal PA (2002). Mutations of the BRAF gene in human cancer. Nature.

[B2] Rajagopalan H, Bardelli A, Lengauer C, Kinzler KW, Vogelstein B, Velculescu VE (2002). Tumorigenesis: RAF/RAS oncogenes and mismatch-repair status. Nature.

[B3] Yuen ST, Davies H, Chan TL, Ho JW, Bignell GR, Cox C, Stephens P, Edkins S, Tsui WW, Chan AS, Futreal PA, Stratton MR, Wooster R, Leung SY (2002). Similarity of the phenotypic patterns associated with *BRAF *and *KRAS *mutations in colorectal neoplasia. Cancer Res.

[B4] Oliveira C, Pinto M, Duval A, Brennetot C, Domingo E, Espin E, Armengol M, Yamamoto H, Hamelin R, Seruca R, Schwartz S (2003). *BRAF *mutations characterize colon but not gastric cancer with mismatch repair deficiency. Oncogene.

[B5] Deng G, Bell I, Crawley S, Gum J, Terdiman J, Allen B, Truta B, Sleisenger M, Kim Y (2004). *BRAF *mutation is frequently present in sporadic colorectal cancer with methylated hMLH1, but not hereditary nonpolyposis colorectal cancer. Clin Cancer Res.

[B6] Nagasaka T, Sasamoto H, Notohara K, Cullings HM, Takeda M, Kimura K, Kambara T, MacPhee DG, Young J, Leggett BA, Jass JR, Tanaka N, Matsubara N (2004). Colorectal cancer with mutation in *BRAF*, *KRAS*, and wild-type withrespect to both oncogenes showing different patterns of DNA methylation. J Clin Oncol.

[B7] Yang S, Farraye FA, Mack C, Posnik O, O'Brien MJ (2004). *BRAF *and *KRAS *Mutations in hyperplastic polyps and serrated adenomas of the colorectum: relationship to histology and CpG island methylation status. Am J Surg Pathol.

[B8] McGivern A, Wynter CV, Whitehall VL, Kambara T, Spring KJ, Walsh MD, Barker MA, Arnold S, Simms LA, Leggett BA, Young J, Jass JR (2004). Promoter hypermethylation frequency and *BRAF *mutations distinguish hereditary non-polyposis colon cancer from sporadic MSI-H colon cancer. Fam Cancer.

[B9] Miyaki M, Iijima T, Yamaguchi T, Kadofuku T, Funata N, Mori T (2004). Both *BRAF *and *KRAS *mutations are rare in colorectal carcinomas from patients with hereditary nonpolyposis colorectal cancer. Cancer Lett.

[B10] Domingo E, Laiho P, Ollikainen M, Pinto M, Wang L, French AJ, Westra J, Frebourg T, Espin E, Armengol M, Hamelin R, Yamamoto H, Hofstra RM, Seruca R, Lindblom A, Peltomaki P, Thibodeau SN, Aaltonen LA, Schwartz S (2004). BRAF screening as a low-cost effective strategy for simplifying HNPCC genetic testing. J Med Genet.

[B11] Toyota M, Ahuja N, Ohe-Toyota M, Herman JG, Baylin SB, Issa JP (1999). CpG island methylator phenotype in colorectal cancer. Proc Natl Acad Sci USA.

[B12] Hawkins NJ, Ward RL (2001). Sporadic colorectal cancers with microsatellite instability and their possible origin in hyperplastic polyps and serrated adenomas. J Natl Cancer Inst.

[B13] Jass JR, Whitehall VL, Young J, Leggett BA (2002). Emerging concepts in colorectal neoplasia. Gastroenterology.

[B14] Chan TL, Zhao W, Leung SY, Yuen ST, Cancer Genome Project (2003). *BRAF *and *KRAS *mutations in colorectal hyperplastic polyps and serrated adenomas. Cancer Res.

[B15] Kambara T, Simms LA, Whitehall VL, Spring KJ, Wynter CV, Walsh MD, Barker MA, Arnold S, McGivern A, Matsubara N, Tanaka N, Higuchi T, Young J, Jass JR, Leggett BA (2004). *BRAF *mutation is associated with DNA methylation in serrated polyps and cancers of the colorectum. Gut.

[B16] Koinuma K, Shitoh K, Miyakura Y, Furukawa T, Yamashita Y, Ota J, Ohki R, Choi YL, Wada T, Konishi F, Nagai H, Mano H (2004). Mutations of *BRAF *are associated with extensive *hMLH1 *promoter methylationin sporadic colorectal carcinomas. Int J Cancer.

[B17] Samowitz WS, Sweeney C, Herrick J, Albertsen H, Levin TR, Murtaugh MA, Wolff RK, Slattery ML (2005). Poor survival associated with the *BRAF *V600E mutation in microsatellite-stable colon cancers. Cancer Res.

[B18] Kawakami K, Ruszkiewicz A, Bennett G, Moore J, Watanabe G, Iacopetta B (2003). The folate pool in colorectal cancers is associated with DNA hypermethylation and with a polymorphism in methylenetetrahydrofolate reductase. Clin Cancer Res.

[B19] Hawkins N, Norrie M, Cheong K, Mokany E, Ku SL, Meagher A, O'Connor T, Ward R (2002). CpG island methylation in sporadic colorectal cancers and its relationship to microsatellite instability. Gastroenterology.

[B20] van Rijnsoever M, Grieu F, Elsaleh H, Joseph D, Iacopetta B (2002). Characterisation of colorectal cancers showing hypermethylation at multiple CpG islands. Gut.

[B21] Ruszkiewicz A, Bennett G, Moore J, Manavis J, Rudzki B, Shen L, Suthers G (2002). Correlation of mismatch repair genes immunohistochemistry and microsatellite instability status in HNPCC-associated tumors. Pathology.

[B22] Wang C, van Rijnsoever M, Grieu F, Bydder S, Elsaleh H, Joseph D, Harvey J, Iacopetta B (2003). Prognostic significance of microsatellite instability and Ki-ras mutation type in stage II colorectal cancer. Oncology.

[B23] Iacopetta B, Elsaleh H, Grieu F, Joseph D, Sterrett G, Robbins P (2000). Routine analysis of p53 mutation in clinical breast tumor specimens using fluorescence-based polymerase chain reaction and single strand conformation polymorphism. Diagn Mol Pathol.

